# Over expression of *METRN* predicts poor clinical prognosis in colorectal cancer

**DOI:** 10.1002/mgg3.1102

**Published:** 2019-12-20

**Authors:** Xin Xu, Chihao Zhang, Yan Xia, Jiwei Yu

**Affiliations:** ^1^ Department of General Surgery Shanghai Ninth People’s Hospital School of Medicine Shanghai Jiao Tong University Shanghai China

**Keywords:** colorectal cancer, GEO, Meteorin, prognosis

## Abstract

**Background:**

The role of meteorin (*METRN*) in colorectal cancer has not been reported previously. We aimed to explore the relationship between *METRN* and colorectal cancer (CRC) prognosis.

**Methods:**

Data were retrieved from the Gene Expression Omnibus database. Gene expression values were log2 transformed and normalized by quantile normalization. Missing values were imputed with the R impute package. Differentially expressed genes were analyzed using the R limma package. *METRN* expression was compared between normal and CRC tissues and among different stages and subtypes of CRC. We assessed the relationship between *METRN* and *KRAS/BRAF* mutations in CRC. Five‐year overall (OS), disease‐free (DFS), and disease‐specific survival (DSS) rates were determined by Kaplan–Meier analysis and analyzed by log‐rank test.

**Results:**

*METRN* was expressed at a higher level in CRC (*p* = .0011) than in normal tissues, especially in advanced stages (*p* = .0343). *METRN* expression levels were higher in the MSI (dMMR) subtype (*p* < .001) and usually with *BRAF* mutations (*p* < .0001). *METRN* overexpression was associated with poor prognosis and low OS (*p* = .01014), DFS (*p* = .0146), and DSS (*p* < .0001) rates.

**Conclusion:**

*METRN* overexpression is a predictive factor for poor prognosis in patients with CRC.

## Graphical abstract

Overexpression of meteorin (METRN) is associated with colorectal cancer and can predict poor prognosis of colorectal cancer.

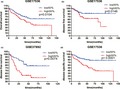

## 1 INTRODUCTION

Colorectal cancer (CRC) is the third most common malignant tumor globally, and more than 1.3 million people are diagnosed with CRC each year (Allemani et al., 2018). It is well known that uncontrolled replication of medial colorectal epithelial cells is an important risk factor for CRC (Dziki et al., 2015). The symptoms of patients with CRC may vary from abnormal weight reduction to changes in defecation habits, due to the different stage, location, and size of tumors. Despite improvements in the diagnosis and treatment of CRC in recent years, the 5‐year survival rates of patients with CRC remain unsatisfactory. For advanced CRC, 5‐year survival rates have been reported to be less than 50% (Liu et al., 2019; Magaji, Moy, Roslani, & Law, 2017). Although an increasing number of biomarkers have been reported to predict CRC, prognostic indicators for patients with CRC remain limited.

Meteorin (*METRN*, OMIM number: 610,998) is a neurotrophic factor with angiogenic properties. It was first described in 2004 (Nishino et al., 2004). Previous studies have shown that *METRN* is mainly expressed in the nervous system, where it plays an important protective role (Jorgensen et al., 2009; Lee, Han, Lee, Park, & Kim, 2010; Wang et al., 2012). *METRN* prevents striatal neurons from becoming excitotoxic, and it reverses motor deficits (Jorgensen et al., 2011). It also plays an antihyperalgesia role in a chronic constriction injury rat model (Xie, Qu, Munro, Petersen, & Porreca, 2019). In some studies, however, *METRN* has been shown to be associated with adverse effects in humans. For example, the serum *METRN* levels are significantly upregulated in pregnant women and can help predict pre‐eclampsia (Garces et al., 2015). In addition, *METRN* has been reported to exert a regulatory function on the progression of various diseases. In recent years, *METRN* and the meteorin‐like (*METRNL*) protein, which is homologous to *METRN*, have been implicated as biomarkers of hematological and endocrine diseases. Nevertheless, there are few studies on the role of *METRN* in CRC, and the relationship between *METRN* expression and CRC patients’ prognosis remains unclear. Therefore, to explore the role of *METRN* in CRC, we performed a retrospective analysis using data from the Gene Expression Omnibus (GEO) database. We found that *METRN* expression was upregulated in CRC and the overexpression of *METRN* was associated with poor prognosis of patients with CRC. Based on these results, we suggest that *METRN* may be used as a biomarker for the prognosis of CRC.

## 2 MATERIALS AND METHODS

### 2.1 Editorial policies and ethical considerations

This study was approved by the Institutional Ethical Review Board of Shanghai Ninth People's Hospital, School of Medicine, Shanghai Jiao Tong University.

### 2.2 Data acquisition and collection

CRC patient data and RNA‐seq expression data were downloaded from the Gene Expression Omnibus (GEO, http://www.ncbi.nlm.nih.gov/geo/). The following search strategy was used: (metrn OR meteorin) AND (colorectal OR cancer OR tumor OR tumour OR carcinoma). We downloaded the original data from the GEO database datasets, http://www.ncbi.nlm.nih.gov/geo/query/acc.cgi?acc=GSE23878, http://www.ncbi.nlm.nih.gov/geo/query/acc.cgi?acc=GSE33113, http://www.ncbi.nlm.nih.gov/geo/query/acc.cgi?acc=GSE17536, http://www.ncbi.nlm.nih.gov/geo/query/acc.cgi?acc=GSE14333, http://www.ncbi.nlm.nih.gov/geo/query/acc.cgi?acc=GSE13067, http://www.ncbi.nlm.nih.gov/geo/query/acc.cgi?acc=GSE13294, http://www.ncbi.nlm.nih.gov/geo/query/acc.cgi?acc=GSE39582, and http://www.ncbi.nlm.nih.gov/geo/query/acc.cgi?acc=GSE37892.

The GenBank reference sequence of *METRN* is Chromosome 16—NC_000016.10, and the version number is 109.20190905.

### 2.3 Statistical analysis

R software was used for data analysis. We used the affy package (R language) to read and process these data. Data were log2 transformed, and the missing values were filled in with the impute package. Differentially expressed genes were filtered out using the limma package. Probes were matched to their corresponding genes using annotation files. We compared the expression levels of *METRN* between CRC tissues and normal tissues in the http://www.ncbi.nlm.nih.gov/geo/query/acc.cgi?acc=GSE23878 dataset. *METRN* expression was evaluated in different Dukes’ stages in the http://www.ncbi.nlm.nih.gov/geo/query/acc.cgi?acc=GSE14333 and http://www.ncbi.nlm.nih.gov/geo/query/acc.cgi?acc=GSE17536 datasets. *METRN* expression levels were also determined in the microsatellite instability (MSI) and microsatellite stability (MSS) subtypes in the http://www.ncbi.nlm.nih.gov/geo/query/acc.cgi?acc=GSE13067 and http://www.ncbi.nlm.nih.gov/geo/query/acc.cgi?acc=GSE13294 datasets, respectively. The association of *METRN* expression level with the histopathological grading of tumors was also analyzed in the http://www.ncbi.nlm.nih.gov/geo/query/acc.cgi?acc=GSE17536 dataset. Moreover, the relationship between *METRN* expression level and *BRAF* and *KRAS* mutations was analyzed in the http://www.ncbi.nlm.nih.gov/geo/query/acc.cgi?acc=GSE39582 dataset. In addition, overall survival (OS), disease‐free survival (DFS), and disease‐specific survival (DSS) curves were generated using the Kaplan–Meier method, and these data were analyzed for statistical significance using a log‐rank test in the http://www.ncbi.nlm.nih.gov/geo/query/acc.cgi?acc=GSE17536 and http://www.ncbi.nlm.nih.gov/geo/query/acc.cgi?acc=GSE37892 datasets. In the above analyses, *p*‐values less than 0.05 indicated statistical significance.

## 3 RESULTS

### 3.1 *METRN* was expressed at a high level in CRC tissues

**Figure 1:**
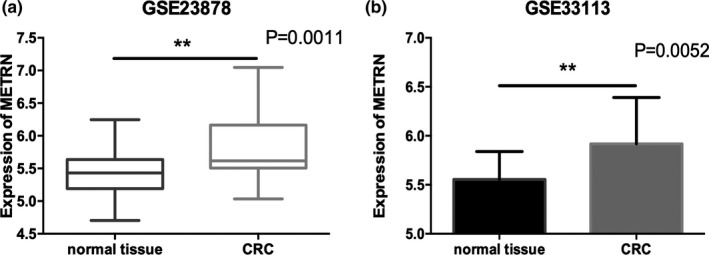
*METRN* expression in normal tissues and colorectal cancer tissues (a‐b). *METRN* GenBank reference sequence: Chromosome 16—NC_000016.10, version number: 109.20190905

We compared the expression of *METRN* between CRC tissues and normal tissues and found that *METRN* was expressed at significantly higher levels in CRC tissues (Figure 1a, b).

### 3.2 *METRN* expression was higher in advanced colorectal cancer

**Figure 2:**
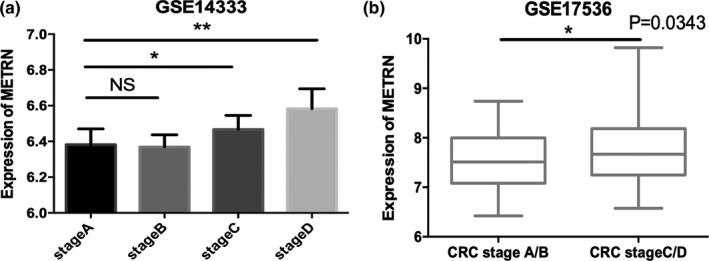
Comparison of *METRN* expression according to Dukes’ stage (a) and early and advanced colorectal cancer (b). NS, not significant. *METRN* GenBank reference sequence: Chromosome 16—NC_000016.10, version number: 109.20190905

We compared the expression of *METRN* in different stages of CRC. We found that *METRN* expression levels were higher in Dukes’ stage C and D tumors, compared with Dukes’ stage A and B tumors (Figure 2a). *METRN* expression levels were not significantly different between CRC stages A and B or between stages C and D. Therefore, we further divided the data into two groups and compared the differences between stage A/B and stage C/D. We found a significant increase in *METRN* expression in the latter group compared with the former group (Figure 2b).

### 3.3 *METRN* was upregulated in the MSI subtype group

**Figure 3:**
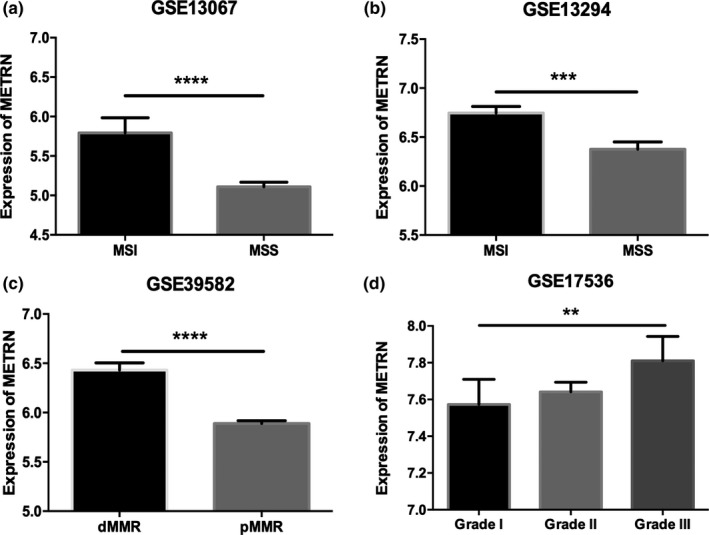
Comparison of *METRN* expression between microsatellite instability and microsatellite stability subtypes (a‐c) and different colorectal cancer grades (d). *METRN* GenBank reference sequence: Chromosome 16—NC_000016.10, version number: 109.20190905

We compared the expression level of *METRN* in MSI and MSS CRC subtypes. We found that *METRN* expression was higher in the MSI subtype than the MSS subtype (Figure 3a, b). *METRN* expression was upregulated to a greater extent in deficient mismatch repair (dMMR) CRC than in proficient mismatch repair (pMMR) CRC (Figure 3c). Moreover, *METRN* expression levels were higher in grade III CRC than in grade I or grade II CRC (Figure 3d).

### 3.4 *METRN *overexpression was associated with *BRAF* mutations

**Figure 4:**
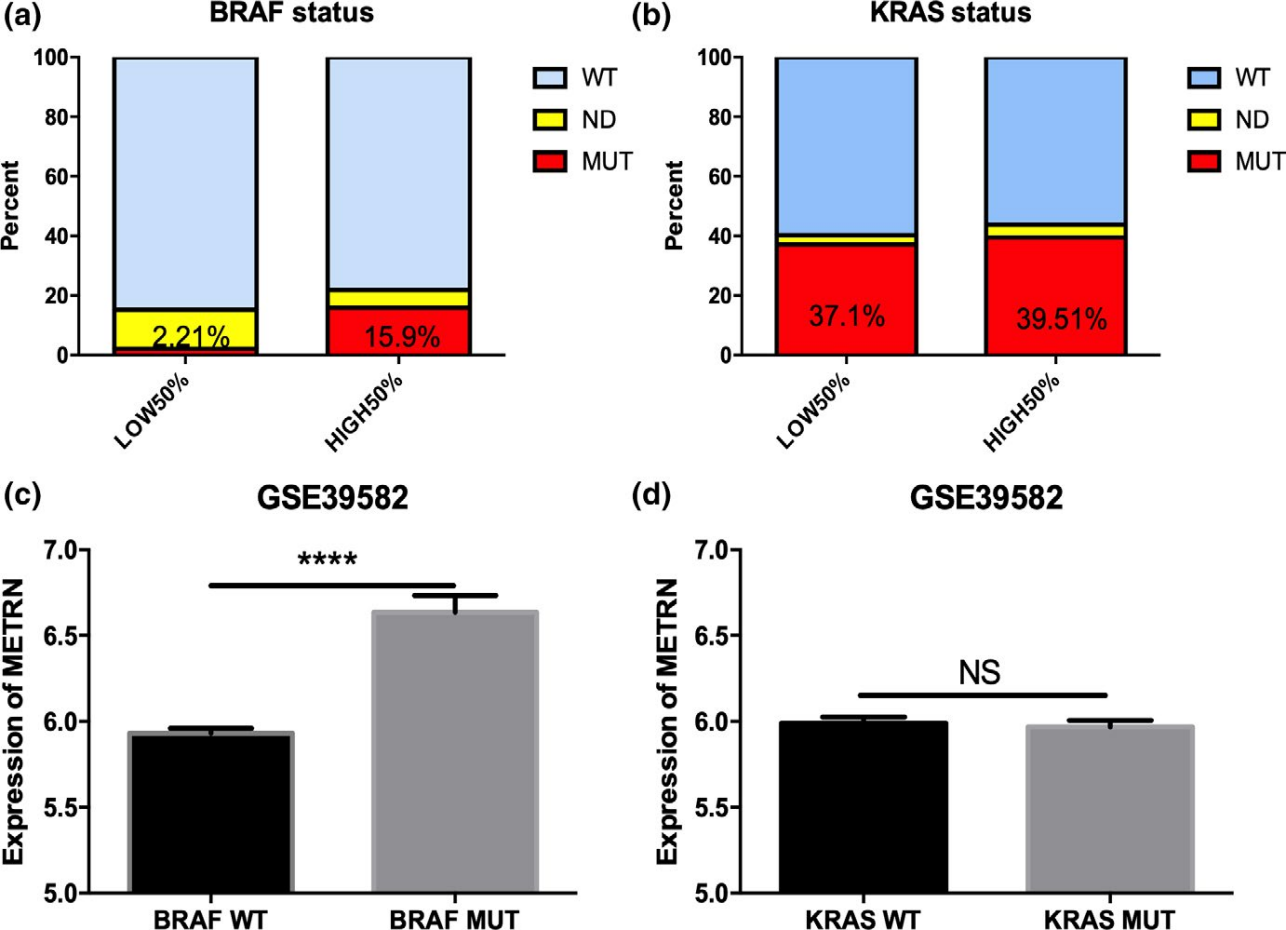
The relationship between *METRN* expression and *BRAF/KRAS* mutations (a and b), the expression of *METRN* in the *BRAF* and *KRAS* mutation‐containing CRC groups (c and d). NS, not significant. WT, wild‐type. MUT, mutation. ND, not determined. *METRN* GenBank reference sequence: Chromosome 16—NC_000016.10, version number: 109.20190905

The *BRAF* mutation rate was significantly higher in the group with high *METRN* expression levels than in the group with low *METRN* expression levels (Figure 4a), while the mutation rate of *KRAS* was not significantly correlated with *METRN* expression level (Figure 4b). *METRN* was expressed at a high level in CRC with *BRAF* mutations (Figure 4c), but not in CRC with *KRAS* mutations (Figure 4d).

### 3.5 *METRN* was associated with poor prognosis in patients with CRC

**Figure 5:**
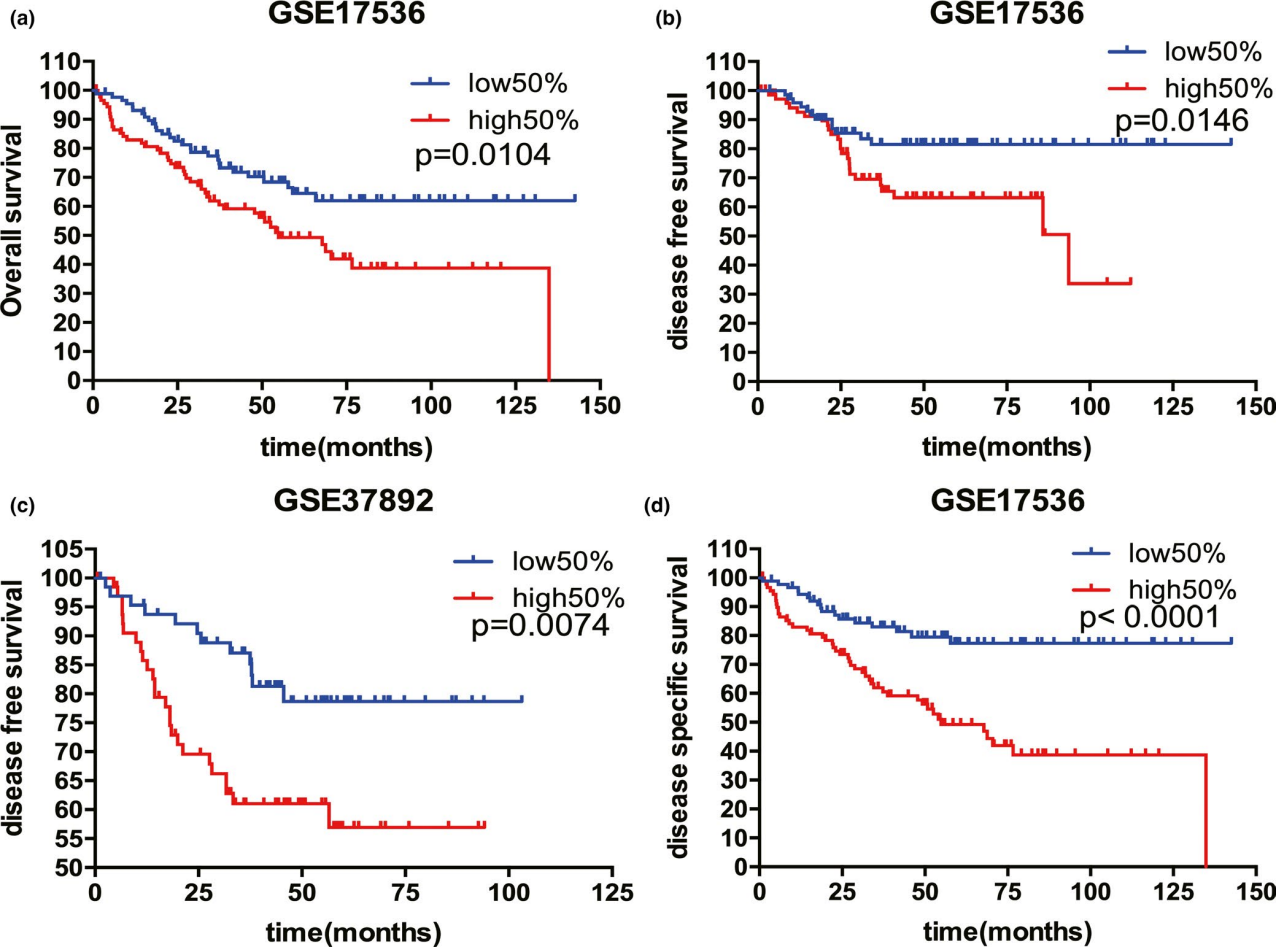
*METRN* expression was associated with overall, disease‐free, and disease‐specific survival (a‐d). *METRN* GenBank reference sequence: Chromosome 16—NC_000016.10, version number: 109.20190905

A Kaplan–Meier curve was plotted to visualize the 5‐year OS, DFS, and DSS rates of patients with CRC. High levels of *METRN* expression were associated with worse 5‐year OS, DFS, and DSS rates in patients with CRC (Figure 5a‐d).

## 4 DISCUSSION

In this study, we found that *METRN* played a critical role in CRC and can be used as a prognostic indicator in patients with CRC. We found that *METRN* was expressed at a high level in CRC tissues compared with normal colorectal tissues. Park et al. previously showed that *METRN* is involved in the regulation of cerebral angiogenesis (Park et al., 2008). Angiogenesis is important for the growth of tumor cells. Advanced tumors often have a rich vascular base. Blocking angiogenesis pathways has been shown to be an effective strategy to improve prognosis in several types of cancer patients (Chan, 2016). *METRN* may be able to block the angiogenic activity of microvascular endothelial cells by inducing the expression of thrombin‐sensitive protein‐1/‐2 in astrocytes (Park et al., 2008). In addition, we found that *METRN* was mainly expressed in Dukes’ stages C and D, which are stages that are usually associated with poor prognosis and low survival rates. To the best of our knowledge, most previous studies on *METRN* have focused on its effects on the central nervous system (Jorgensen et al., 2009; Kim et al., 2014; Wang et al., 2012), and it has rarely been studied in CRC. This is the first study showing the significance of *METRN* in human CRC, indicating that *METRN* may perform a critical role in the prognosis of patients with CRC.

CRC can be classified into the MSI and MSS subtypes, according to the microsatellite stability. The extent of mismatch repair (MMR) can be used as the basis of risk stratification. There is ample evidence that patients with dMMR have a lower risk of recurrence and longer survival time than those with pMMR (Sargent et al., 2010). MSI is the molecular marker for dMMR, and it is often associated with improved overall survival rates in early‐stage CRC. High‐level MSI (MSI‐high) occurs in approximately 15% of patients with CRC and is associated with improved survival rates (Popat, Hubner, & Houlston, 2005; Roth et al., 2012; Vilar & Tabernero, 2013). MSI CRC is more prone to somatic mutations than the MSS or pMMR subtypes of CRC (Chong et al., 2019). In this study, we found that *METRN* expression was upregulated in the MSI CRC group. This result may indicate that a higher incidence of somatic mutations occurs in CRC with high *METRN* expression. It is interesting to note that *METRN* was expressed at a higher level in the CRC group with *BRAF* mutations than in the group with *KRAS* mutations. *KRAS* and *BRAF* are common sites of somatic mutations in many types of human cancers, especially CRC (Yuen et al., 2002). Mutations in both these genes can increase mortality in CRC patients with both MSS‐ and MSI‐type tumors (Lochhead et al., 2013; Phipps et al., 2015). Recent studies have shown that MSI‐type colorectal tumors exhibit a relatively low frequency of *BRAF* and *KARS* mutations (Febbo et al., 2011; Funkhouser et al., 2012; Ogino, Kawasaki, Kirkner, Loda, & Fuchs, 2006). Yuen et al. showed that *BRAF* mutations are associated with early Dukes’ tumor stages in CRC (Yuen et al., 2002). However, Garcia et al. reported that *BRAF* mutations are associated with poorer differentiation, mucinous histology, MSI, and larger primary tumors (Sanz‐Garcia, Argiles, Elez, & Tabernero, 2017). Yaeger et al. found that metastatic CRCs with *BRAF* mutations were prone to progress from stage III disease, and T4 disease was more common in patients with stage III disease (Yaeger et al., 2014). Consequently, we are more inclined to conclude that *BRAF* mutations indicate poor prognosis. Further histopathological analysis of *METRN* expression in CRC confirmed our conclusion.

In survival analysis, *METRN* was found to be a significant predictor of OS, DSS, and DFS, with rates of survival being negatively correlated with *METRN* expression. This suggested that *METRN* expression is a significant risk factor in patients with CRC. A previous study reported that *METRNL* could alleviate lipid‐induced inflammation and insulin resistance via *AMPK* or PPARδ‐dependent pathways (Jung et al., 2018). Some studies have shown that the *AMPK* and PPARδ signaling pathways play important roles in CRC. *AMPK* has an important autophagy‐inducing effect during the treatment of tumors, including CRC tumors, and this can be regulated by redox modification under oxidative stress (Kim, Kundu, Viollet, & Guan, 2011; Yan et al., 2019). Furthermore, it has been reported that PPARδ induces CRC metastasis and increases the activity of CRC cells through various pathways (Wang, Fu, Wei, Xiong, & DuBois, 2019; Zhou, Jin, Liu, Shi, & Hou, 2019). Therefore, we propose that *METRN* may be involved in regulating CRC prognosis through the above mechanisms.

In addition, it has been reported that low serum *METRNL* levels may be associated with endothelial dysfunction (El‐Ashmawy, Selim, Hosny, & Almassry, 2019). Therefore, we propose that *METRN* expression may be involved in regulating the function of colorectal endothelial cells. Another study found that *METRN* is highly expressed in early embryos during gastrulation and is crucial for mesoendoderm development (Kim et al., 2014). This further increases the evidence that *METRN* regulates the function of colorectal endothelial cells.

Although our study revealed that *METRN* might be a prognostic indicator in patients with CRC, we did not explore the possible mechanism of *METRN*’s involvement in CRC regulation. Therefore, we cannot completely confirm the specific role of *METRN* in CRC. Additional studies are recommended to explore the role of *METRN* in CRC. Since there are no previous reports on the relationship between *METRN* and CRC, our results will play a guiding role for future studies.

In conclusion, overexpression of *METRN* was closely associated with advanced CRC stage and predicted poor clinical outcomes. Therefore, our results may have a more detailed reference value for the prognosis of patients with CRC in the future clinical practice.

## CONFLICT OF INTEREST

The authors have no conflict of interest to declare.
